# Pulmonary Arterial Hypertension and Chronic Thromboembolic Pulmonary Hypertension: An Immunological Perspective

**DOI:** 10.3390/jcm9020561

**Published:** 2020-02-19

**Authors:** Thomas Koudstaal, Karin A. Boomars, Mirjam Kool

**Affiliations:** Department of Pulmonary Medicine, Erasmus MC, Doctor Molenwaterplein 40, 3015 GD Rotterdam, The Netherlands; k.boomars@erasmusmc.nl (K.A.B.); m.kool@erasmusmc.nl (M.K.)

**Keywords:** pulmonary arterial hypertension, chronic thromboembolic pulmonary hypertension, inflammation and immunity, circulating inflammatory biomarkers, immunomodulatory therapy

## Abstract

Pulmonary hypertension (PH) is a debilitating progressive disease characterized by increased pulmonary arterial pressures, leading to right ventricular (RV) failure, heart failure and, eventually, death. Based on the underlying conditions, PH patients can be subdivided into the following five groups: (1) pulmonary arterial hypertension (PAH), (2) PH due to left heart disease, (3) PH due to lung disease, (4) chronic thromboembolic PH (CTEPH), and (5) PH with unclear and/or multifactorial mechanisms. Currently, even with PAH-specific drug treatment, prognosis for PAH and CTEPH patients remains poor, with mean five-year survival rates of 57%–59% and 53%–69% for PAH and inoperable CTEPH, respectively. Therefore, more insight into the pathogenesis of PAH and CTEPH is highly needed, so that new therapeutic strategies can be developed. Recent studies have shown increased presence and activation of innate and adaptive immune cells in both PAH and CTEPH patients. Moreover, extensive biomarker research revealed that many inflammatory and immune markers correlate with the hemodynamics and/or prognosis of PAH and CTEPH patients. Increased evidence of the pathological role of immune cells in innate and adaptive immunity has led to many promising pre-clinical interventional studies which, in turn, are leading to innovative clinical trials which are currently being performed. A combination of immunomodulatory therapies might be required besides current treatment based on vasodilatation alone, to establish an effective treatment and prevention of progression for this disease. In this review, we describe the recent progress on our understanding of the involvement of the individual cell types of the immune system in PH. We summarize the accumulating body of evidence for inflammation and immunity in the pathogenesis of PH, as well as the use of inflammatory biomarkers and immunomodulatory therapy in PAH and CTEPH.

## 1. Introduction

Pulmonary hypertension (PH) is a devastating condition characterized by increased pulmonary vascular resistance and elevated pulmonary arterial pressure. The definition of PH has recently been updated and is currently defined as increased mean pulmonary arterial pressures (PAP) above 20 mmHg at rest [1]. This enhanced pulmonary arterial pressure leads to elevated right ventricular (RV) hypertrophy, heart failure and, ultimately, death [2]. Structural remodeling of the vasculature, leading to diameter reduction, is linked to increased pulmonary vascular resistance and increased pulmonary pressure. PH severity is indicated by the New York Heart Association (NYHA) functional classification (FC), ranging from no symptoms (Class I) to severe symptoms at rest (Class IV).

Based on the underlying causes of PH, the WHO classification system divides PH patients into 5 groups: (1) pulmonary arterial hypertension (PAH), (2) PH due to left heart disease, (3) PH due to lung disease, (4) chronic thromboembolic PH (CTEPH), and (5) PH with unclear and/or multifactorial mechanisms [1]. WHO Group 1 (PAH), which is defined as a condition with a mean PAP >20 mmHg, normal left atrium pressure and pulmonary vascular resistance ≥3 Wood units [1], consisting of different subgroups based upon different underlying diseases or pathophysiological mechanisms. Heritable PAH (HPAH) includes patients with a family history or identified germline mutation. Such heritable susceptibility is conveyed not only through mutations in the bone morphogenetic protein receptor type II (*BMPR2*) gene, but also through newly identified mutations in novel causal genes [3]. PAH can also be induced by specific drugs and toxins [4]. Other causes of PAH are called APAH (associated PAH); PAH due to congenital heart disease (CHD), mainly comprising a ventricle septum defect, an atrial septum defect and patent ductus arteriosus; PAH due to liver disease (porto-pulmonary PAH), PAH due to HIV and PAH due to schistosomiasis. PAH can also be associated with auto-immune diseases, specifically with systemic sclerosis (SSc, prevalence 10%–15%), mixed connective tissue diseases (MCTD) and systemic lupus erythematosus (SLE). Two more separate identities in PAH WHO Group 1 are pulmonary veno-occlusive disease (PVOD), which can be heritable (eukaryotic translation initiation factor 2α kinase *EIF2AK4* mutation), associated with autoimmune diseases or idiopathic, and persistent pulmonary hypertension of the newborn (PPHN). PPHN can be idiopathic or may be caused by several pulmonary diseases. In the largest group of PAH; idiopathic PAH (IPAH), no cause or associated disease is identified so far. WHO group 4 patients (CTEPH) can be further differentiated by whether they are operable (eligible for pulmonary endarterectomy (PEA) or balloon pulmonary angioplasty (BPA)) or inoperable CTEPH.

Currently, PAH-specific drugs focus predominantly on dilatation of the pulmonary arterial vasculature [5]. In inoperable CTEPH, PAH-specific drugs are also used to modulate the increased pulmonary vascular pressure [2]. However, even with PAH-specific drug treatment, survival for PAH patients remains poor with mean five-year survival rates of 57%–59% [6,7] and 53%–69% for PAH and inoperable CTEPH, respectively [7,8,9]. Therefore, more insight into the pathogenesis of PAH and CTEPH is urgently needed, so that new therapeutic strategies can be developed.

Especially in PAH, an increasing body of evidence shows that inflammation might play a role in its pathobiology [10]. However, recent studies have demonstrated that inflammatory cells might also contribute to disease pathology in CTEPH [11,12]. This review aims to summarize the expanding knowledge about inflammatory cells in the pathogenesis of PH, as well as the use of inflammatory biomarkers and immunomodulatory therapy in PAH and CTEPH.

## 2. Inflammation and Immunity in PAH and CTEPH

### 2.1. Histopathology in PAH and CTEPH

**Figure 1:**
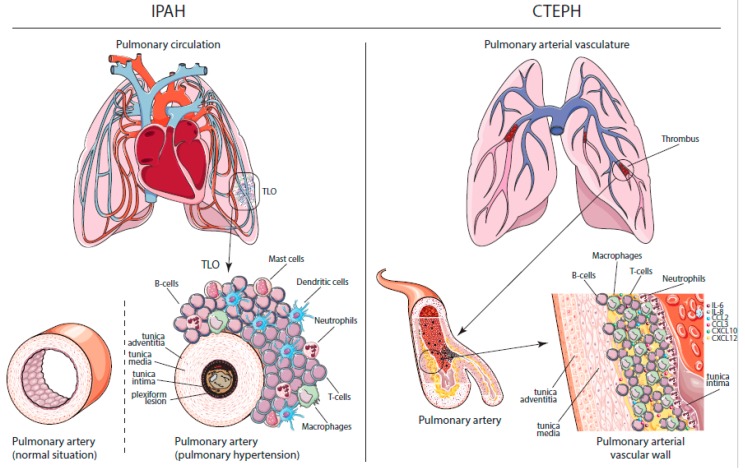
Immunohistopathology in IPAH and CTEPH. Left: schematic overview of cells involved in tertiary lymphoid organs (TLOs) in idiopathic pulmonary arterial hypertension (IPAH) patients. In the pulmonary hypertension (PH) situation, endothelial hyperproliferation is visible in the tunica intima with plexiform lesion formation in the lumen of the artery. Furthermore, smooth muscle cell (SMC) hyperplasia is visible in the tunica media of the pulmonary artery. Surrounding the tunica adventitia is a combination of B cells, T cells, mast cells, dendritic cells, neutrophils and macrophages. Right: schematic overview of vascular remodeling and inflammation in the thrombotic material of chronic thromboembolic pulmonary hypertension (CTEPH) patients. Between the (neo)intimal vascular wall and the tunica media, an influx of inflammatory cells such as B cells, T cells, neutrophils and macrophages is visible. Moreover, the enhanced presence of pro-inflammatory mediators, such as interleukin (IL)-6, IL-8, chemokine (C-C motif) ligands 2 and 3 (CCL2 and CCL3), C-X-C motif chemokines 10 and 12 (CXCL10 and CXCL12) is present.

PAH and CTEPH are characterized by vascular remodeling as a result of increased pulmonary arterial pressures (Figure 1). Features of pulmonary vascular remodeling in PH are intima wall thickening and the formation of obliterative concentric lesions in the endothelial and/or smooth muscle cell layers. In the media, which mainly consists of smooth muscle cells, an increase in thickness is also seen. Interestingly, the combined intima and media thickness correlated significantly to the PAP and the pulmonary vascular resistance (PVR) [13]. Finally, increased adventitial thickness and remodeling were the most prominent findings in a series of 19 IPAH patient lung autopsies [14]. However, this increased adventitial thickness was not confirmed in a recent study, which might be explained by methodological differences between these studies [13].

Besides increased intimal, medial and adventitial vascular thickness, another prominent feature in PAH patients is perivascular inflammation. A feature seen specifically in IPAH patients is the formation of plexiform lesions, which are typically defined as dynamic networks of vascular channels formed by monoclonal proliferation of endothelial cells [15]. In CTEPH patients, histologic studies show neointimal, thrombotic, recanalized and atherosclerotic lesions in the pulmonary arterial vasculature. These chronic thrombotic lesions contain collagen, elastin, inflammatory cells, re-canalization vessels and calcification [16]. Current concepts state that CTEPH is a dual vascular disorder with stenosis, webs and occlusions in large- and medium-sized pulmonary arteries (Pas) at the sites of previous pulmonary emboli. Furthermore, a secondary arteriopathy affecting small resistance vessels is visible in lung biopsies of CTEPH patients [17].

### 2.2. Dysregulated Immune Responses in PAH and CTEPH

Immune responses can be divided into two categories—innate and adaptive immunity. Innate immunity is our swift first-line defense against microorganisms and foreign pathogens, which is very broad and nonspecific. Innate immune responses are usually initiated and executed by macrophages, neutrophils, natural killer (NK) cells and dendritic cells (DCs). In parallel, adaptive immune responses are initiated, which allows for more specific responses executed by T and B lymphocytes. Next to their innate function, DCs initiate adaptive immune responses by migrating to lymphoid organs, mostly the tissue-draining lymph nodes. There, they present parts of foreign pathogens on their cell surface through major histocompatibility complex (MHC) molecules in order to induce the clonal expansion of antigen-specific T cells. Memory T cells and B cells can ensure lifelong immunity, allowing our body to rapidly and more vigorously respond to pathogens upon a second encounter. The adaptive response relies on the ability of T and B cells to distinguish self from non-self antigens. If this capacity is disturbed, the immune system may attack the body’s own cells, which can lead to autoimmune disease. Elements hereof can be found in both PAH and CTEPH. IPAH, for instance, is characterized by perivascular inflammation, consisting of pulmonary lymphoid neogenesis with the formation of tertiary lymphoid organs (TLOs), which contain specific T and B cell zones with on-site activation and production of antibodies [18]. In addition to lymphocytes, these inflammatory lesions also contain DCs, macrophages and mast cells [18,19]. Moreover, there is a positive correlation between the degree of pulmonary perivascular inflammation and vascular intimal/medial/adventitial thickness and mean PAP, suggesting that this inflammation could be involved in pulmonary vascular remodeling and PH development [20].

In CTEPH patients, thrombotic and atherosclerotic lesions were found to contain activated B and T lymphocytes, macrophages, and neutrophils [21]. In this study, topographic analyses revealed a transmural distribution of T cells, whereas B cells were low in number and mostly localized deep within the lesion, close to internal elastic lamina and native media. Although inflammatory cells are common in thrombotic material, the accumulation of inflammatory cells may be a sign of involvement in the pathology of the non-resolution of thrombosis and atherosclerosis in CTEPH patients.

Below, we discuss several different immune cells that are part of the innate or adaptive immune system and their possible roles in the pathogenesis of PH.

#### 2.2.1. Innate Immunity

##### Macrophages

Macrophages are first-line myeloid leucocytes observed in pulmonary lesions in PH patients [22,23]. Pulmonary macrophages can classically be divided into interstitial and alveolar macrophages, although recent single-cell RNA sequencing and lineage tracing studies have defined multiple pulmonary macrophage subtypes in mice [24,25,26]. In a hypoxia-driven PH mouse model, specifically interstitial macrophages—but not alveolar macrophages—were increased [27]. They might be involved in PH development through their production of cytokines [28,29,30]. Furthermore, the macrophages present in lung perivascular spaces in PAH are derived from peripheral blood monocytes [31], likely indicating their recruitment and differentiation due to pulmonary inflammation. However, chemokines, such as chemokine (C-C motif) ligand 2 (CCL2) and chemokine (C-X3-C motif) ligand 1 (CX3CL1) monocytes are recruited to the site of the inflammation and differentiate into inflammatory macrophages. In PH, patients’ expression of chemokines, such CCL1, CCL2, CCL5 and CX3CL1, was increased in circulatory monocytes, along with increased pulmonary levels of CCl1, CCL2, CCL3, CLL3 and CX3CL1 [31]. In CX3CR1 (receptor for CX3CL1)-deficient mice, both pulmonary inflammation and vascular remodeling were reduced after exposure to hypoxia when compared to control mice [31]. Interestingly, in pulmonary vascular endothelial cells from PAH patients, excessive expression of CCL2 was observed, which acts as a chemoattractant for circulating inflammatory cells and as a growth factor for pulmonary arterial smooth muscle cells (PASMCs). Moreover, PASMCs and perivascular macrophages from patients with PAH exhibited elevated CCR2 and CCR5 levels compared to controls [32,33]. Indeed, expression of CCR2 and CCL5-CCR5 was needed in both macrophages and PASMCs to initiate and amplify PASMC proliferation [33]. When circulating monocytes differentiate into interstitial macrophages in hypoxia-induced PH in mice, they express thrombospondin-1 (TSP-1), leading to Rho kinase-mediated vasoconstriction through transforming growth factor beta (TGF-β) activation [34], thereby amplifying PH pathology. All these studies suggest a potentially crucial role for chemokine-mediated macrophage recruitment in the early pathogenesis of PH.

Macrophage recruitment and activation was also shown to play an important role in the pathogenesis of hypoxia-induced PH in the mouse and in Sugen/athymic rat models [35,36,37]. A previous study has reported that hypoxia-inducible factor-1*α* (HIF-1*α*) is expressed by pulmonary macrophages in PH patients, especially in plexiform lesions [38]. When HIF-1*α* was absent in mice, a significant reduction in right ventricular (RV) systolic pressure (RVSP) and RV hypertrophy was observed, together with less infiltration of macrophages in the lung and RV, indicating the direct effect of on-site macrophages in inducing PH [39]. In IPAH patients, increased numbers of macrophages and monocytes were found in the lungs when compared to healthy controls [40]. Another PH-inducing factor, resistin-like molecule-α (RELMα) was increased in a hypoxia-induced mouse model for PH, and its human homologue, resistin, was also found to be upregulated in macrophage-like inflammatory cells in IPAH patients. In these patients, resistin-stimulated macrophages promoted the apoptosis-resistant proliferation of PASMCs [41].

Macrophages may also play a role in other forms of PH, as the increased presence of inflammatory macrophages was apparent in surgical pulmonary endarterectomy (PEA) material from CTEPH patients [21]. Moreover, in serum from eight CTEPH patients, increased expression of macrophage inflammatory protein-1α (CCL3) was detected, which can lead to the synthesis of inflammatory cytokines, vascular remodeling and recruitment of macrophages [42]. Recently, the accumulating evidence for the role for macrophages in PH has been extensively reviewed by Florentin et al. and Pullamsetti et al. [43,44]. Taken together, macrophages play a pivotal role in the pathogenesis of PH, through the production of inflammatory cytokines, initiation and proliferation of PASMCs and hypoxia factors.

##### Neutrophils

Neutrophils are early responders and are recruited to sites of acute inflammation, in response to chemokines produced by tissue-resident immune cells, such as macrophages. Neutrophils are known phagocytes, capable of ingesting microorganisms or particles. Besides their phagocytic role, neutrophils are able to degranulate and release antimicrobial contents. In PAH patients, an increased neutrophil-to-lymphocyte ratio in peripheral blood samples positively correlates with the NYHA FC and a negative ratio can even predict event-free survival [45,46]. In CTEPH patients, the neutrophil-to-lymphocyte ratio could predict postoperative mortality and might be used as a noninvasive measuring tool for operative risk stratification [47]. Currently, it is unclear if and how neutrophils contribute to PAH progression. In murine models, neutrophils accumulated at the site of inflammation/injury in the lungs of hypoxic PH and in monocrotaline (MCT)-induced PH rats [36,48]. Recent evidence suggests that the neutrophilic production of myeloperoxidase (MPO), a catalyst for reactive oxygen species (ROS) formation, can cause disease progression [49]. Plasma levels of MPO were found to be increased in PAH patients compared to healthy controls. Furthermore, hypoxia-exposed *Mpo*^-/-^ mice showed a lower increase in RV pressure than wildtype mice [49], indicating a pathogenic role for neutrophils in PH through the production of MPO and adverse pulmonary vascular function.

##### Mast Cells

Mast cells (MCs) are long-living tissue-resident immune cells known for their important role in the immune system through their release of histamine and production of inflammatory cytokines. MCs are also known for their role in angiogenesis through their production of vascular endothelial growth factor (VEGF) and MC proteases, including chymase and tryptase [50,51,52]. Accumulating evidence indicates a role for MCs in the pathophysiology of PH. MCs are present in inflammatory lesions in IPAH patients [19], even in early perivascular cellular lesions in the lungs of IPAH patients [53]. In recent years, the role of MCs has been shown in proof-of-principle experiments. In *Ws/Ws* rats, in which MCs are absent due to a mutation in mast cell growth factor receptor c-kit [54], features of experimental PH, such as RVSP, PVR, RV hypertrophy and vascular remodeling were largely attenuated after pulmonary arterial banding or MCT treatment [55]. Furthermore, when degranulation of MCs was inhibited by ketotifen, the development of PH was reduced in several experimental rat PH models [55,56,57,58]. In a small clinical trial, nine PAH patients were treated with the MC inhibitors cromolyn and fexofenadine. In these patients, a decrease in VEGF levels and circulating proangiogenic myeloid cells was observed, together with an increase in exhaled nitric oxide (which is generally low in PAH), indicating this treatment might have a suppressive effect on MCs [59]. Mechanistically, MC proteases are believed to play an important role in the process of PH development and severity. MC proteases, such as chymase and tryptase, measured in the lung tissue, correlate with the severity of PH and pulmonary vascular remodeling [52,60,61,62,63]. Moreover, excessive MC infiltration and degranulation was detected in the lung tissue pf MCT-rats and not in the RV, indicating a release of proteases, such as tryptase, which contribute to pulmonary vascular remodeling [64]. Lastly, MCs are observed around distal pulmonary arteries, together with accumulated macrophages, in MCT-challenged rats, suggesting that MCs are involved in vascular remodeling in the lungs [64]. In summary, MCs are involved in PH pathobiology, most likely through the release of proangiogenic factors and MC proteases.

##### Natural Killer Cells

Natural killer (NK) cells comprise an important part of the innate immune system, as they provide rapid responses to virus-infected cells, but these cytotoxic cells are also known for regulating angiogenesis and vascular remodeling. Few studies have evaluated the possible contribution of NK cells in the pathogenesis of PAH and CTEPH. However, in both PAH patients and in rodent experimental PH models, impaired NK cell numbers and cytotoxicity were found [65]. Furthermore, in two independent genetic mouse models for NK cell dysfunction, involving deficiency of the NFIL3 transcription factor or the NK activating receptor NKp46, enhanced RV systolic pressures and RV hypertrophy were found. In both models, this experimental PH development was linked to increased interleukin-23 (IL-23) production, possibly due to NK cells or impairment, leading to the increased production of IL-23 by pulmonary macrophages and other myeloid cell types [66]. Interestingly, IL-23 is known for its production of inflammatory cytokines, such as IL-17A/F, IL-21 and IL-22, and for driving naïve T cells to a TH17 phenotype [67]. Taken together, NK cell defects may contribute to PAH pathogenesis by the aberrant regulation of pulmonary vascular remodeling, however further research is required to evaluate these findings.

#### 2.2.2. Linking Innate and Adaptive Immunity

##### Dendritic Cells

DCs are key modulators between tolerance and immunity and are known to function as a bridge between innate and adaptive immunity. The main function of DCs is to capture, process and present antigens to T cells. DCs can be activated either by microbial stimuli through pattern recognition receptors, such as Toll-like receptors, or by inflammatory cytokines, which leads to the activation of the nuclear factor kappa-light-chain-enhancer of activated B cells (NF-κβ) pathway. Next, DCs upregulate costimulatory molecules, produce various inflammatory cytokines, such as interleukin (IL)-6 and IL-12, and, together with antigen presentation, DCs promote T-cell activation, expansion and differentiation [68,69,70]. This process must be tightly controlled, as continuous DC activation could lead to severe side-effects, such as the presentation of self-antigens to T-cells, resulting in development of auto-immune diseases [69,71]. Rationally, DCs could also be involved in the pathophysiology of PAH [72].

In IPAH patients, DCs are increased in the lung, specifically accumulating around remodeled pulmonary arteries [73]. However, in the parenchyma, mostly immature DCs are observed, shown by an increased number of dendritic cell-specific intercellular adhesion molecule 3-grabbing non- integrin (DC-SIGN)^+^ DCs [73]. DCs can be divided into four main subtypes: type I conventional DCs (cDC1s), which are efficient at cross-presentation (i.e., presentation of exogenous antigens in the context of MHC Class I and elicitation of CD8+ T-cell responses); type II cDCs (cDC2s), which are capable of inducing CD4+ T-cell responses; plasmacytoid DCs (pDCs), which can produce large amounts of type I interferons to combat viral infections; and, finally, monocyte-derived DCs (mo-DCs) that arise during inflammation and produce large amounts of chemokines, attracting T-cells to the site of inflammation [74].

The numbers of cDCs and pDCs were increased both in total lung cell suspensions (either peripheral or perihilar samples) and in the larger pulmonary arteries of IPAH patients compared to controls [40]. Confocal microscopy analyses showed that pDCs were predominantly localized in the alveolar space, in proximity to blood vessels. In contrast, in peripheral blood, cDC numbers were decreased in IPAH patients [75] and, together with the increase in pulmonary cDCs, this suggests migration to the lungs. Currently, no data is available for different cDC subsets in the pathogenesis of PAH. Taken together, DCs are crucial in initiating adaptive immune responses and could be involved in the pathogenesis of PAH by antigen presentation and production of inflammatory cytokines.

#### 2.2.3. Adaptive Immunity

##### T Cells

T cells are a vital part of the adaptive immune system. CD4+ T cells or T-helper (Th) cells, provide help by indirectly killing pathogens by supporting the activation of other cells in the immune system, such as B cells. CD8+ T cells are known as ‘cytotoxic T-cells’, which are able to directly kill pathogens through the release of granzymes, which induce apoptosis, and the pore-forming protein perforin, which creates holes in target-cell membranes. Gamma delta (γδ) T cells represent a small subset of T cells which are defined by the expression of heterodimeric T-cell receptors (TCRs) composed of γ and δ chains. An increased number of CD4+, CD8+ T-cells and γδ T cells was found in close proximity to the pulmonary arteries in IPAH lung biopsies using flow cytometry [40]. These CD4+ and CD8+ T cells are present in the adventitial space around the pulmonary vessels in IPAH patients [19]. In schistosomiasis-associated PAH and IPAH patients, increased peri-arterial CD4+ T cells were found as well [76].

Th cells are known to play an important role in many inflammatory and autoimmune diseases [77]. Th-cells can be roughly divided into Th1, Th2 and Th17 cells. Especially, Th17 cells are found in the pulmonary TLOs of IPAH patients [18]. Th17 cells are the main source of IL-17, IL-21, and IL-22. In remodeled PAs of IPAH patients, IL-21^+^ cells are present [78]. Th17 cells differentiate from naïve Th-cells in the presence of IL-1β, IL-6, and TGF-β [79]. In serum, both IL-1β and IL-6 are increased in IPAH patients compared to controls [80]. In CTD-PAH, Th17 cells and Th17-related cytokines were increased when compared to the healthy controls [81]. Work from our group has shown that the level of tumor necrosis factor alpha (TNF-α)-induced protein 3 (TNFAIP3) expression in DCs controls T-cell differentiation, because TNFAIP3-deficient DCs promote Th17-cell differentiation through increased expression of IL-1β, IL-6 and IL-23 [71,82]. DC-specific deletion of the *TNFAIP3* gene also leads to increased NF-kB, creating a pro-inflammatory environment.

Follicular T-helper (Tfh) cells, expressing the CXCR5 chemokine receptor which contributes to their localization in B cell follicles, can support activated B cells under the influence of IL-21, IL-6, IL-12 and IL-27, leading to the induction of humoral immune responses. In IPAH patients’ TLOs, an increase was found in IL-21^+^ PD1^+^ Tfh cells [18]. In CTEPH patients, the histological evaluation of PEA material showed accumulation of CD3+ T cells in atherosclerotic and thrombotic lesions [21]. Little is known about T-cell function differences in CTEPH and more research is needed to provide evidence for a possible pathogenic role for T cells in CTEPH pathogenesis. Taken together, T cells are increased in IPAH lungs and CTEPH PEA material. It appears that a dysregulated Th17-immune response is present in PAH; however, more studies are needed to further elaborate this.

##### B Cells and Humoral Immune Responses

B cells are the effectors of the humoral immune response. Following antigen recognition by the B cell receptor they can present antigens, secrete cytokines and differentiate into memory B cells or plasma cells that produce large amounts of antibodies. Upon activation, B and T cells engage in a germinal center reaction, in which Tfh cells produce their canonical cytokine IL-21, which supports B cell survival, proliferation and differentiation. Moreover, activated T cells express CD40L, which interacts with its receptor CD40 in B cells to provide a co-stimulatory signal that is critical for B cell activation and germinal center formation. As mentioned above, the lungs of PAH patients contain TLOs containing B cells, T cells and DCs. These highly organized structures contain high endothelial venules, enabling circulating lymphocytes and stromal cells, including follicular dendritic cells that present antigens to B cells via Fc-receptors, to directly enter [83]. Importantly, the presence of IL-21+ Tfh cells, B cells that express activation-induced cytidine deaminase that is essential for immunoglobulin heavy chain class switch and antibody affinity maturation, and plasma cells, provide evidence for local and ongoing antibody production [18]. Next to the presence of TLOs in the lung, there is additional evidence supporting the notion that B-cell activation is dysregulated in IPAH and in connective tissue disease associated PAH (CTD-PAH) [84,85]. First, circulating plasma blasts are increased in IPAH patients [86]. Second, autoantibodies are present in approximately 40% of patients with IPAH [87]. These autoantibodies might be produced by the plasma cells located within TLOs in IPAH lungs [18,86], recognizing endothelial cell surface antigens [88]. Anti-endothelial autoantibodies promote apoptosis of endothelium, which contributes to vascular remodeling [86]. Furthermore, endothelial-specific IgA can promote cytokine production and the upregulation of adhesion molecules by endothelial cells [86,88,89,90]. Anti-endothelial IgG antibodies activate endothelial cells to a pro-adhesive and pro-inflammatory state [91]. In animal studies, the injection of autoantibodies from CTD-PAH patients into healthy mice leads to more abundant vascular and airway smooth muscle cell numbers and inflammatory pulmonary vasculopathy [92]. In MCT rats, high levels of plasma IgG were found that labeled lung vascular proteins. Moreover, the transfer of autoantibodies into rats caused pulmonary vascular remodeling and pulmonary hypertension [93].

In CTEPH, little is known about circulating and thrombus-resident B-cells. A well-known risk factor for CTEPH is a splenectomy and, considering that the spleen is important for B cell maturation, there might be a role for pathogenic B-cells in the CTEPH pathogenesis. Currently, studies are being performed using mass cytometry in PBMCs from CTEPH patients. Results from these studies are still in progress.

In summary, autoantibodies are found in IPAH patients, specifically targeting endothelial cell surface antigens. B-cells and plasma cell formation prior to this could play a major role in the pathogenesis of PAH.

## 3. Inflammatory Diagnostic and Prognostic Biomarkers in PAH and CTEPH

Inflammatory biomarkers might be useful as diagnostic and prognostic tools in PAH and CTEPH [21,32,42,80,94,95,96,97,98,99,100,101,102]; these are highlighted in Table 1. Inflammatory cytokines and chemokines can contribute directly to the recruitment of immune cells, the activation and proliferation of PASMCs, and endothelial dysfunction. Until now, the most prominent cytokine appears to be IL-6, which has many links to PAH pathogenesis. In animal models, PH development has been seen after the administration of recombinant IL-6 and also in IL-6 transgenic mice [103,104,105], whereas IL-6 knockout mice have shown resistance to hypoxia-induced PH development [106]. In clinical studies, IL-6 has shown to correlate to survival and quality of life in IPAH patients [80,107] and in predicting long-term responses to PEA in CTEPH patients [98]. In a recent study, cytokine clusters were made in PAH patients using machine learning. The analyses showed that the immune phenotypes were not dependent upon the subtypes within the WHO Group 1 PAH classification [108], indicating that immune phenotypes may vary within the WHO Group 1 subtypes. Furthermore, these findings might provide a framework to examine patient responses to emerging therapies targeting immunity in the future.

In tumor necrosis factor alpha (TNF-α)-overexpressing mice, the spontaneous development of PH was observed [109]. In a clinical study in CTEPH patients undergoing PEA, increased levels of TNF-α, IL-6 and IL-10 were found prior to surgery [97]. IL-6 and IL-10 were shown to peak immediately after surgery, while TNF-α decreased significantly within the first 24 h after PEA surgery [97].

In CTD-PAH, treatment-naïve patient baseline levels of placental growth factor (PlGF), sVEGFR-1, TNF-α, and VEGF-D were increased and could differentiate between healthy controls/IPAH and CTD-PAH. Moreover, after four months of PAH-targeted treatment, sVEGFR-1 levels were decreased, indicating that this growth factor is worthwhile to evaluate during therapies [99]. In a study of 206 PAH patients, angiopoietin 1 (Ang-1), VEGF and matrix metallopeptidase 9 (MMP-9) levels have been associated with increased risk of death and hospitalization at the 16-week follow-up point after baseline [101]. Many of the inflammatory biomarkers are still being investigated, because more pre-clinical, translational and clinical studies are needed to determine the clinical and prognostic value of these markers.

## 4. Immunomodulatory Therapy in PAH and CTEPH

**Figure 2:**
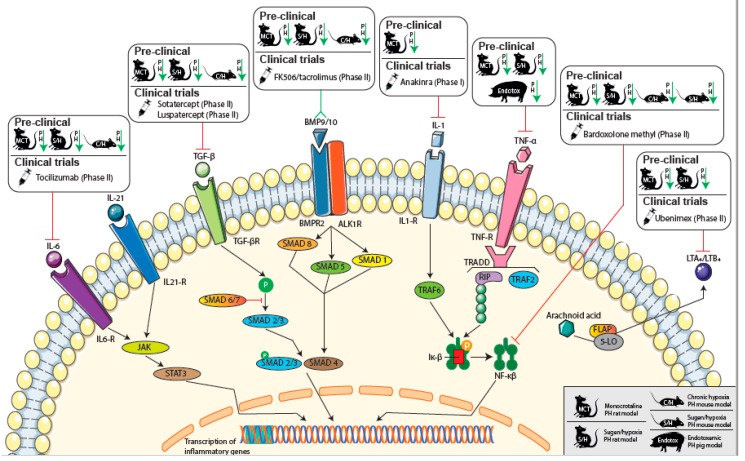
Immunomodulatory therapeutic targets in PAH. Schematic overview of (pre-)clinical targets for immunomodulatory therapy in pulmonary arterial hypertension (PAH) patients. Pre-clinical animal PH model signs are mentioned in the legend in the bottom right corner. Interleukin (IL); transforming growth factor beta (TGF-β); bone morphogenetic protein (BMP); bone morphogenetic protein receptor type II (BMPR2); tumor necrosis factor-a (TNF-α); nuclear factor kappa-light-chain-enhancer of activated B cells (NF-κβ,); Leukotriene A4/B4 (LTA4/B4). Janus kinases (JAK); signal transducer and activator of transcription proteins (STAT); small mothers against decapentaplegic (SMAD); TNF receptor-associated factor (TRAF); tumor necrosis factor receptor type 1-associated death domain (TRADD); receptor-interacting protein (RIP)**;** inhibitor of κβ (Iκ-β); 5-lipoxygenase activating protein (FLAP); arachidonate 5-lipoxygenase (5-LO).

Promising novel inflammatory therapeutic targets and ongoing clinical trials evaluating possible therapeutic drug compounds are highlighted in Figure 2. In addition, an overview of the attenuation of experimental PH by targeting immunomodulatory pathways is given in Supplementary Table S1.

Based on the evidence described above, targeting immune and inflammatory pathways may be sufficient to treat and prevent progression of the disease. Previous studies have shown that in the inflammatory MCT-rat PH model, anti-inflammatory therapies, such as dexamethasone, mycophenolate mofetil and the nuclear factor of activated T cell (NFAT) inhibition with cyclosporine can prevent and reverse the PH phenotype [110,111,112]. In SLE and MCTD-PAH patients, treatment with a combination of cyclophosphamide and glucocorticoids was possibly effective in lowering the PVR in patients with a less severe PH at baseline [113].

More targeted therapy, such as anti-IL-1 treatment was shown to prevent the PH phenotype in MCT-PH rats [114]. A current trial, conducted by the Virginia Commonwealth University, evaluating the efficacy of treatment of PAH patients with anakinra, an IL-1 receptor antagonist compound, will soon be finalized.

In recent studies, IL-6-specific antagonist treatment reversed experimental PH in MCT-PH and sugen/hypoxia (SU/Hx)-induced PH rat models [115]. In hypoxia-induced PH in mice, the attenuating effect of a blockade of IL-6 was also found [78]. Currently, the TRANSFORM-UK trial is running, in which PAH patients are being treated with anti-IL-6, and results of this study are expected soon [116].

In hypoxia-induced PH mice, increased expression levels were found for IL-17 and IL-21, signature genes for Th17 and Tfh cells, respectively [78]. Whereas the blockade of IL-17 showed no effects on the RVSP and the RV hypertrophy, IL-21-receptor knockout mice were resistant to hypoxia-induced PH [78]. Increased expression of M2 macrophage markers and IL-21, which can polarize macrophages towards an M2 phenotype, was detected in the lungs of IPAH patients who underwent lung transplantation. Together with the known prominent role of Il-21 in B-T cell interaction, these findings suggest that IL-21 is a potential target for treating PAH [78]. In autoimmune experimental arthritis, a combination of IL-6/IL-21 blockades have shown synergistic beneficial effects associated with strongly reduced Th17 differentiation [117].

Anti-TNF-α therapy (etanercept) has been demonstrated to attenuate the PH phenotype both in MCT-PH rats [118,119] as well as in SU/Hx-induced PH rats [120]. In endotoxemic pigs, anti-TNF-α therapy reversed the PAH phenotype [121]. Currently, no (pre-)clinical trials are available to determine the possible clinical effects of etanercept in patients.

Increasing knowledge on loss-of-function mutations in the BMPR2 signaling pathway have led to the initiation of studies evaluating possible novel therapeutic targets in this cascade. BMPR2, which is mainly expressed in vascular endothelial cells [122], is a member of the TGF-β receptor family and many studies have shown an important role for BMPR2 in the pathogenesis of PAH. Upon binding to bone morphogenetic proteins (BMPs), BMPR2 initiates intracellular signaling that ultimately leads to the inhibition of proliferation of vascular smooth muscle tissue. In smooth muscle cells, BMP signaling can be directly inhibited by TGF-β signaling and involved ligands are able to function as antagonists in competition for type II receptor binding [123]. *BMPR2* loss-of-function mutations are a known cause for PAH development in patients and lead to more severe disease and increased risk of death when compared to PAH patients without a *BMPR2* mutation [124]. In a recent study, BMP9 was shown to be a sensitive and specific biomarker of porto-pulmonary hypertension patients in order to predict transplant-free survival and the presence of PAH in liver disease [125]. In human IPAH lungs and in hypoxia-induced PH in mice, reduced BMPR2 expression-induced macrophage recruitment—involving enhanced production of the chemokine granulocyte macrophage colony-stimulating factor (GM-CSF)—led to the exacerbation of PAH features [126]. In rodent models, such as the MCT-rat, Su/Hx PH mouse model and in mice harboring a human *BMPR2* mutation knock-in allele, BMPR2 activation can prevent vascular remodeling and can attenuate the PAH phenotype with endothelial growth and proliferation [127,128,129]. Recently, a therapeutic drug discovery company (MorphogenIX, Cambridge, UK) was founded for the development of BMPs as a novel treatment for PAH.

Targeting TGF-β signal pathways may also be an effective treatment for PAH, considering the upregulation of TGF-β downstream of the loss of function in BMPR2, which has shown a correlation with PH development [130,131]. In a recent study, treatment with immunoglobulin-Fc fusion protein TGF-β (TGFBRII-Fc), a selective TGF-β inhibitor targeting THF-b 1/3, has shown attenuation of the PH phenotype in MCT-PH rats and SU/Hx-induced PH rats and mice [132]. Currently, ligand traps, such as Sotatercept and Luspatercept, with high selectivity for members of the TGF-β superfamily are being investigated in phase II trials (PULSAR trial), as they were successful in previous phase I clinical trials. These ligand traps may rebalance BMPR2 signaling and restore vascular homeostasis.

Another potentially interesting compound in the BMPR2 pathway is calcineurin inhibitor FK506 (tacrolimus), which has been reported to increase the expression and activity of BMPR2. In MCT-PH and SU/Hx-induced PH rats and hypoxia-induced PH mice, FK506 was reported to reverse the severe PAH phenotype [133]. In IPAH patients, FK506 treatment reversed dysfunctional BMPR2 signaling in the pulmonary artery endothelial cells [133]. In a recent phase IIa trial, treatment with FK506 in 20 PAH patients showed increased expression of BMPR2, improvement of 6MWD and the serological and echocardiographic parameters of heart failure in some patients; however, these changes were not significant [134]. Nonetheless, FK506 was generally well tolerated and this study supports the initiation of a phase IIb efficacy trial. Significantly, tacrolimus is also well known for other immunosuppressive effects, such as T cell inhibition in the organ-transplant field, so the effects might not be exclusively limited to the BMPR2 pathway.

The nuclear factor kappa-light-chain-enhancer of activated B cells (NF-κβ) is a ubiquitous transcription factor that is known for the regulation of many aspects of innate and adaptive immune functions. By inducing expression of various pro-inflammatory genes for cytokines and chemokines, NF-κβ is an important regulator for cell survival, proliferation and mobility [135]. Targeting NF-κβ may therefore be an interesting novel therapeutic pathway for the treatment of PAH. In MCT-PH rats, the NF-κβ was found to be activated and an NF-κβ-blocking treatment attenuated the PH phenotype [136,137,138]. This ameliorating effect was also found in SU-Hx rats, in which NF-κβ targeting severely reduced lung vascular lumen obliteration [139]. Furthermore, in MCT-treated transgenic mice overexpressing a cardiac-specific dominant-negative inhibitory binding partner of NF-κβ (IκBαP), the inhibition of NF-κβ prevented right ventricular hypertrophy (RVH) [140]. In PAH patients, NF-κβ has been shown to be highly activated in pulmonary lymphocytes, macrophages, endothelial cells and PASMCs [141].

Currently, several NF-κβ inhibitory compounds are available for the evaluation of treatment efficacy in PH patients. Bardoxolone methyl is a known inhibitor of NF-κβ and shows effects in suppressing the activation of pro-inflammatory mediators, enhancement of endothelial NO bioavailability, improvement of metabolic dysfunction, suppressing vascular proliferation and preventing maladaptive remodeling [142,143,144]. Currently, bardoxolone methyl is being evaluated in a phase II clinical trial in pulmonary hypertension patients (IPAH, CTD-PAH, WHO group III or group V PH).

Another promising compound is dimethyl fumarate (DMF), with potent anti-inflammatory effects through its inhibition of NF-κβ. DMF is an activating agent for the transcriptional regulator nuclear factor erythroid 2-related factor 2 (NRF2), which is known for its key regulation of antioxidant genes [145]. NRF2 function is linked to NFκB signaling with activation of NRF2, leading to inhibition of NFκB signaling and thereby inducing an anti-inflammatory response [146]. In the chronic hypoxia and SU/Hx-induced PH mouse model, DMF has been reported to attenuate the PAH phenotype [147]. Currently, no clinical trial for the evaluation of the clinical therapeutic value of DMF is being performed.

As previously described, accumulating evidence is beginning to show a pathological role for B cells and plasma cells in PH pathogenesis. Therefore, targeting CD20, which is a B-cell specific surface marker, could be a promising drug therapy to evaluate. In a case report, rituximab treatment significantly improved early onset PAH in a young patient suffering from SLE [148]. Currently, the National Institute of Allergy and Infectious Diseases (NIAD) is conducting a phase II prospective, double-blind, placebo-controlled, multi-center, randomized trial for evaluating the effect of treatment with rituximab in patients suffering from SSc-PAH.

Another interesting therapeutic target is leukotriene B4 (LTB_4_), a pro-inflammatory lipid mediator produced from arachidonic acid by the consecutive activities of 5-lipoxygenase, 5-lipoxygenase-activating protein, and leukotriene A_4_ hydrolase [149]. In MCT-PH rats, the LTB_4_ receptor antagonist (ONO4057) has been reported to reduce RVH after MCT treatment and prevent development of PH [150].

Nonspecific inhibition of Leukotriene A4 through bestatin (ubenimex) reversed the PH phenotype in MCT-PH and SU/Hx-induced PH rats [37]. Currently, a clinical phase II trial (LIBERTY trial) is evaluating the efficacy of bestatin treatment in 61 PAH patients. Preliminary results for the trial sponsor (Eiger Biopharmaceuticals, Palo Alto, CA, USA), however, show no significant treatment effect in comparison to placebo treatment.

## 5. Summary

Taken together, there is mounting evidence that the immune system plays a pivotal role in the pathogenesis of PAH and CTEPH. Both PAH and CTEPH histology demonstrated the extensive accumulation of immune cells. Further analyses in IPAH patient lungs and lung biopsy material from CTEPH patients provided compelling evidence for the activation of the innate immune system. The pathological involvement of macrophages, MCs and neutrophils by production of inflammatory cytokines, recruitment of other immune cells and local inflammation and damage was demonstrated. In the lungs of IPAH patients, increased numbers of DCs were observed, acting as a bridge between the innate and adaptive immune system by the presentation of antigens to T cells. DCs contribute to increased production of cytokines and chemokines, attracting other inflammatory cells to the site of inflammation. Dysregulated Th17 immunity was found in PAH patients, creating a pro-inflammatory auto-immune environment. Moreover, IPAH patients displayed an increase in circulating autoantibodies specifically targeting endothelial cell surface antigens. Extensive biomarker research revealed that many inflammatory and immune markers correlate with the hemodynamics and/or prognosis of PAH and CTEPH patients. However, further evaluation is required to investigate the applicability of these parameters in the clinical work-up of PAH and CTEPH patients. Currently, clinical trials are being performed to assess the value of promising inflammatory and immune targets defined in pre-clinical research in PAH. A combination of immunomodulatory therapies might be required besides current treatment based on vasodilatation alone, to establish the effective treatment and prevention of progression of this disease.

## Figures and Tables

**Table 1:** Circulating cytokine/chemokine Levels in patients with pulmonary arterial hypertension or chronic thromboembolic pulmonary hypertension.

	PAH						CTEPH			
Biomarker	Incident Patients	Prevalent Patients	Hemodynamic Correlation	Prognosis	Etiology	Ref.	Prevalent Patients	Hemodynamic Correlation	Prognosis	Ref.
IL-1α	↑[94]	↑	N/A	+	IPAH, HPAH	[94]				
IL-1β	↑[94]	↑	−	+ [94]	IPAH, HPAH, CHD-PAH	[80,94]	↑[98]	−	−	[21,96,98]
IL-2	=	↑	−	−	PAH, HPAH	[80,94]	↑	−	−	[98]
IL-4	=	↑	−	−	PAH, HPAH	[80,94]	↑	−	−	[98]
IL-5	N/A	=	−	−	PAH, HPAH	[80]	=	−	−	[98]
IL-6	↑[94,95,99]	↑	+	+	IPAH, HPAH, CTD-PAH, CHD-PAH	[80,94,95,99,100]	↑[42,97]	+	+ [97,98]	[21,42,95,97,98]
IL-8	=	↑	−	+ [80]	IPAH, HPAH, CTD-PAH, CHD-PAH	[80,94,95,99,100]	↑[42,97,98]	−	+ [97,98]	[42,95,97,98]
IL-10	=	↑	−	+ [80]	IPAH, HPAH	[80,94]	↑	−	+ [97,98]	[21,97,98]
IL-12	=	↑	−	+ [80]	IPAH, HPAH	[80,94]	=	−	−	[98]
IL-13	=	=	−	+ [94]	IPAH, HPAH	[80,94]	=	−	−	[98]
IFN-γ	=	=	−	−	IPAH, HPAH	[79,93]	=	−	−	[98]
TNF-α	↑[94,95,99]	↑	N/A	+ [94]	IPAH, HPAH, CTD-PAH, CHD-PAH	[80,94,95,99,100]	=	−	+ [96,97]	[95,96,97]
MMP-9	↑	↑	+	+	PAH	[101]	↑	−	N/A	[21]
VEGF	=	↑	N/A	+ [101]	IPAH, HPAH, CHD-PAH	[80,94,100,101]	=	−	N/A	[21]
CCL-2	=	↑[33]	N/A	−	IPAH	[33,94]	↑	−	N/A	[21,42]
MIG	N/A	↑	−	N/A	IPAH	[42]	↑	−	N/A	[42]
CCL-3	=	=	−	N/A	IPAH	[42]	↑	+	N/A	[21,42]
CXCL-10	N/A	↑	−	N/A	IPAH	[42]	↑	+	N/A	[42]
CCL-5	N/A	↑	−	N/A	IPAH, PAH	[42,102]	↓	−	N/A	[42]
CX3CL-1	=	=	−	N/A	IPAH	[42]	=	−	N/A	[42]
CXCL-12	=	=	−	N/A	IPAH	[42]	=	−	N/A	[42]

Pulmonary arterial hypertension (PAH); idiopathic PAH (IPAH); hereditary PAH (HPAH); congenital heart disease-associated PAH (CHD-PAH); connective tissue disease-associated PAH (CTD-PAH); chronic thromboembolic pulmonary hypertension (CTEPH); no significant differences (=); positive correlation (+); no significant correlation (−); not assessed (N/A); interleukin (IL); interferon gamma (IFN-γ); tumor necrosis factor-a (TNF-α); matrix metallopeptidase 9 (MMP-9); vascular endothelial growth factor (VEGF); chemokine (CCL) (C-C motif) ligand; monokine-induced by interferon-γ (MIG); C-X-C motif chemokine (CXCL); C-X3-C motif ligand (CX3CL).

## Supplementary Materials

The following are available online at https://www.mdpi.com/2077-0383/9/2/561/s1, Table S1: Pre-clinical therapeutic targets in experimental PH.

## Abbreviations

Ang-1	Angiopoietin 1
APAH	associated PAH
BPA	Balloon pulmonary angioplasty
*BMPR2*	Bone morphogenetic protein receptor type II
BMPs	Bone morphogenetic proteins
tacrolimus	Calcineurin inhibitor FK506
CCL	Chemokine C-C motif ligand
CXCL	Chemokine C-X3-C motif ligand
CTEPH	Chronic thromboembolic pulmonary hypertension
CHD	Congenital heart disease
CTD-PAH	Connective tissue disease associated PAH
DC-SIGN	Dendritic cell- specific intercellular adhesion molecule-3 grabbing non- integrin
DCs	Dendritic cells
DMF	Dimethyl fumarate
Tfh	Follicular T-helper cells
FC	Functional class
γδ	Gamma delta
HPAH	Heritable PAH
TCRs	Heterodimeric T-cell receptors
HIF-1*α*	Hypoxia-inducible factor-1*α*
IPAH	Idiopathic PAH
IL	Interleukin
LTB_4_	Leukotriene B4
CCL3	Macrophage inflammatory protein-1α
MHC	Major histocompatibility complex
MCs	Mast cells
MMP-9	Matrix metallopeptidase 9
MCTD	Mixed connective tissue diseases
MCT	Monocrotaline
mo-DCs	Monocyte-derived DCs
MPO	Myeloperoxidase
NIAD	National Institute of Allergy and Infectious Diseases
NK	Natural killer cells
NYHA	New York Heart Association
NF-κβ	Nuclear factor kappa-light-chain-enhancer of activated B cells
NFAT	Nuclear factor of activated T cell
Porto-pulmonary PAH	PAH due to liver disease
PPHN	Persistent pulmonary hypertension of the newborn
PlGF	Placental growth factor
pDCs	Plasmacytoid DCs
PAH	Pulmonary arterial hypertension
PAP	Pulmonary arterial pressures
PASMCs	Pulmonary arterial smooth muscle cells
PEA	Pulmonary endarterectomy
PH	Pulmonary hypertension
PVR	Pulmonary vascular resistance
PVOD	Pulmonary veno-occlusive disease
ROS	Reactive oxygen species
RELMα	Resistin-like molecule-α
RV	Right ventricular
RVH	Right ventricular hypertrophy
SU/Hx	Sugen/hypoxia
SLE	Systemic lupus erythematosus
SSc	Systemic sclerosis
TLOs	Tertiary lymphoid organs
Th	T-helper cell
TSP-1	Thrombospondin-1
TNFAIP3	TNF Alpha Induced Protein 3
NRF2	Transcriptional regulator nuclear factor erythroid 2-related factor 2
TGF-β	Transforming growth factor beta
TNF-α	Tumor necrosis factor alpha
cDC1s	Type 1 conventional DCs
cDC2s	Type 2 cDCs
VEGF	Vascular endothelial growth factor
