# Supplementary material for: Pulmonary Arterial Hypertension and Chronic Thromboembolic Pulmonary Hypertension: An Immunological Perspective
Source: J Clin Med. 2020 Feb 19;9(2):561. doi: 10.3390/jcm9020561 (PMC7074374; doi:10.3390/jcm9020561)
Supplement: Supplementary file 1 [file jcm-09-00561-s001.pdf]

Table 1. Pre-clinical therapeutic targets in experimental PH.

Target	Therapy	MCT-PH Rats	Sugen/hypoxia PH rats	Chronic/hypoxia PH mice	Sugen/hypoxia PH mice	Endotoxemic PH pigs	Ref
IL-1	Antagonist	X					114
IL-6	Antagonist	X	X	X			78,115
TGF-β	Antagonist	X	X	X			132
BMPR2	Agonist	X	X	X			127,128,131
TNF-α	Antagonist	X	X			X	118-121
NF-κβ	Antagonist	X	X	X	X		136-139,147
LTA4/B4	Antagonist	X	X				37,150
